# Systemic venous anomalies in the Middle East

**DOI:** 10.3389/fped.2013.00001

**Published:** 2013-02-26

**Authors:** Antonio F. Corno, Sami A. Alahdal, Karuna Moy Das

**Affiliations:** ^1^Pediatric and Congenital Cardiac Surgery, King Fahad Medical CityRiyadh, Kingdom of Saudi Arabia; ^2^Department of Radiology, King Fahad Medical CityRiyadh, Kingdom of Saudi Arabia

**Keywords:** anomalous systemic venous connections, cardiopulmonary bypass, congenital heart defects, geographical distribution, interuption of inferior vena cava, isomerism, persistent left superior vena cava, retro-aortic innominate vein

## Abstract

**Introduction:** Systemic venous anomalies are quite rare and can be associated with congenital heart disease requiring surgery.

**Materials and Methods:** All consecutive patients (pts) undergoing surgery for congenital heart defects were retrospectively analyzed for presence of systemic venous anomalies:

(a) Persistent left superior vena cava (PLSVC)
(b) Inferior vena cava (IVC) interruption
(c) Retro-aortic innominate vein

**Results:** From 9/2010 to 5/2012 155 pts, median age 7 months, mean age 1.3 years (3 days–50 years), median weight 4 kg, mean weight 7.2 kg (0.6–110 kg) underwent congenital heart surgery. Twenty-nine systemic venous anomalies were identified in 28/155 patients (=18.1%). PLSVC was present in 21 pts (=13.5%), median age 4 months, mean age 2.7 years (3 days–22 years), median weight 6 kg, mean weight 10.1 kg (2.4–43.0 kg). IVC interruption was identified in 5 pts (=3.2%), median age 2 months, mean age 5.4 years (30 days–26 years), median weight 3.7 kg, median weight 17 kg (2.3–68.0 kg). Retro-aortic innominate vein was diagnosed in 3 pts (=1.9%), median age 5 years, mean age 3.7 years (10 months–5 years), median weight 12 kg, mean weight 10.1 kg (4.5–14 kg). Complete pre-operative diagnosis was obtained in 14/28 (=50%) pts with echocardiography and in other 8/28 (=28.6%) only after computed tomography (CT) scan, for a total of 22/28 (=78.6%) correct pre-operative diagnosis. In 6/28 (=21.4%) patients the diagnosis was intra-operative. Total incidence of systemic venous anomalies was 18.1% (vs. 4% in the literature, *P* = 0.0009), with presence of PLSVC = 13.5% (vs. 0.3–4.0%, respectively *P* = 0.0004 and *P* = 0.0012), IVC interruption = 3.2% (vs. 0.1–1.3%, N.S.), and retro-aortic innominate vein = 1.9% (vs. 0.2–1%, N.S.).

**Conclusions:** Our study showed an incidence of systemic venous anomalies in Middle Eastern pts with congenital heart defects higher than previously reported. In 78.6% of pts the diagnosis was correctly made before surgery (echocardiography or CT scan), with 21.4% of complete diagnosis made at surgery. A careful pre-operative screening should be performed in all pts with congenital heart defects from this region to better identify all systemic venous anomalies for a more accurate surgical planning.

## Introduction

In patients with congenital heart defects requiring surgery the pre-operative knowledge of associated systemic venous anomalies plays a major role in the appropriate selection of the surgical approach. The detailed description and location of anomalous systemic venous connections are extremely valuable for the decision-making relative to: (a) the choice among the available surgical options; (b) the plan of the surgical technique; (c) the implications relatives to the venous cannulation for cardio-pulmonary bypass.

This single center retrospective study was motivated by the impression of a relative high occurrence of systemic venous anomalies observed in the initial surgical activity of a newly established pediatric and congenital cardiac surgery unit in comparison with the literature reports (1–4).

## Materials and methods

A retrospective analysis was performed on all patients undergone surgery for congenital heart defects from the beginning of the clinical activities in a new unit of pediatric and congenital heart surgery.

The primary diagnosis was established on the basis of the echocardiography findings in all patients, who underwent complete pre-operative echocardiography screening following the institutional protocol. Further supplement of investigation with computed tomography (CT) scan was performed on indication.

The hospital records of all patients with pre-operative echocardiography and (CT) scans were retrospectively reviewed for the presence of one of the following systemic venous anomalies:

(a) Persistent left superior vena cava (PLSVC)
(b) Interruption of the inferior vena cava (IVC)
(c) Retro-aortic innominate vein

The review results were then compared with the reports of the intra-operative findings.

Finally, the incidence of the systemic venous anomalies recorded in our study was statistically compared with the incidence reported in the literature using the two-tailed *T*-test.

## Results

From September 2010 to March 2012, 155 consecutive patients, median age 7 months, mean age 1.3 years (range 3 days to 50 years), median body weight 4 kg, mean weight 7.2 kg (range 0.6–110 kg), underwent surgery for palliation or repair of congenital heart defects.

The catchment areas of all these patients were from Middle Eastern countries: Kingdom of Saudi Arabia, Egypt, Iran, Jordan, Oman, Palestine, Syria, Yemen, and United Arab Emirates.

Systemic venous anomalies were identified in 28/155 patients (=18.1%). One patient presented with association of PLSVC and IVC interruption.

(a) PLSVC was present in 21 patients (=13.5%), median age 4 months, mean age 2.7 years (range 3 days–22 years), median body weight 6 kg, mean weight 10.1 kg (range 2.4–43.0 kg).
(b) IVC interruption was identified in 5 patients (=3.2%), median age 2 months, mean age 5.4 years (range 30 days–26 years), median body weight 3.7 kg, median weight 17 kg (range 2.3–68.0 kg).
(c) Retro-aortic innominate vein was diagnosed in 3 patients (=1.9%), median age 5 years, mean age 3.7 years (range 10 months–5 years), median body weight 12 kg, mean weight 10.1 kg (range 4.5–14 kg).

Pre-operative identification of the systemic venous anomaly was obtained in 22/28 (=78.6%) patients.

Table 1 shows the mode of detection of each of the observed systemic venous anomalies.

**Table 1:** **Mode of detection of the systemic venous anomalies in 28 patients**.

**Type of venous anomaly**	**Echo**	**CT scan**	**Surgery**	**Total**
Persistent left superior VC	13	2	5	20
Inferior VC interruption	1	4	0	5
Retro-aortic innominate vein	0	2	1	3
Total	14 (= 50%)	8 (= 28.6%)	6 (21.4%)	28

VC, vena cava.

Table 2 indicates the congenital heart defects associated with each of the systemic venous anomalies.

**Table 2:** **Associated congenital heart defects**.

**No.**	**Associated defects**
**PLSVC**	
4	Complete Atrio-Ventricular Septal Defect
	1 with Aortic Coarctation, Hypoplastic Aortic Arch
4	Ventricular Septal Defect
	1 with sub-aortic obstruction, RVOTO
3	Tetralogy of Fallot
	3 with Right Aortic Arch
	1 with anomalous coronary artery
3	Double Outlet Right Ventricle, Ventricular Septal Defect
	1 with Pulmonary Atresia, Right Aortic Arch
	1 with Pulmonary Stenosis, Transposition of the Great Arteries
	1 with straddling Tricuspid Valve, Transposition of the Great Arteries
2	Atrial Septal Defect
1	Mitral Stenosis
	1 with Ventricular Septal Defect, Hypoplastic Aortic Arch
1	Double Orifice Mitral Valve
	1 with sub-aortic obstruction, Aortic Coarctation, Hypoplastic Aortic Arch
1	Mitral Atresia
	1 with Pulmonary Stenosis, Transposition Great Arteries, Right Aortic Arch
1	Single Ventricle
	1 with Common Atrium, Pulmonary Stenosis, discontinuity of Pulmonary Arteries
**IVC-I**	
3	Left Isomerism
	3 with univentricular heart, Total Anomalous Pulmonary Venous Connection
1	Total Anomalous Pulmonary Venous Connection
	1 with Ventricular Septal Defect
1	Tetralogy of Fallot
	1 with Partial Anomalous Pulmonary Venous Connection
**RAIV**	
3	Tetralogy of Fallot
	3 with Right Aortic Arch

IVC-I, Inferior Vena Cava Interruption; PLSVC, Persistent Left Superior Vena Cava; RAIV, Retro-Aortic Innominate Vein.

The comparison of the incidence of the systemic venous anomalies observed in this study versus the data available in the literature showed the results indicated in Table 3.

**Table 3:** **Comparison of the observed incidence with the literature**.

**Type of anomaly**	**Reported (References)**	**Observed**	**Statistic**
Total	4.0% (1–15)	18.1% (28/155)	*P* = 0.0009
PLSVC	0.3–4.0% (1–15)	13.5% (21/155)	*P* = 0.0004–0.012
IVC-I	0.1–1.3% (1, 4, 16)	3.2% (5/155)	N.S.
RAIV	0.2–1.0% (17–27)	1.9% (3/155)	N.S.

IVC-I, Inferior Vena Cava Interruption; PLSVC, Persistent Left Superior Vena Cava; RAIV, Retro-Aortic Innominate Vein.

The total incidence of systemic venous anomalies in the Middle Eastern population with congenital heart defects undergoing surgery resulted statistically higher (*P* = 0.0009) than previously reported in the literature. With regard to the incidence of each of the observed anomalies there was a trend toward higher incidence of each systemic venous anomalies, even though it reach statistical significance only for the PLSVC. This could be due to the small number of patients, rendering the study not powered enough to detect a difference at a significant level.

## Discussion

The incidence of systemic venous anomalies reported in the literature is depending upon the modality utilized for identification. These anomalies can be diagnosed by echocardiography, angiography, CT scan, MRI, intra-operative evidence, or finding at autopsy. In general systemic venous anomalies are more frequent in patients with congenital heart defects than in the normal population (1–4).

Since the presence of systemic venous anomalies in patients with congenital heart defects has influence of the surgical decision-making, all the information should be available in the pre-operative phase.

In the initial period of activity of a new unit of pediatric and congenital cardiac surgery, because of the impression that the incidence of systemic venous anomalies in our surgical population was higher than reported in the literature, we prompted this retrospective study.

Our observation was limited to the following systemic venous anomalies:

(a) Persistent left superior vena cava (PLSVC)
(b) Interruption of the inferior vena cava (IVC)
(c) Retro-aortic innominate vein

### Persistent left superior vena cava (PLSVC)

PLSVC has been reported in 0.3–0.5% of the normal population, but up to 4% in patients with congenital heart defects (1–15).

**Figure 1:**
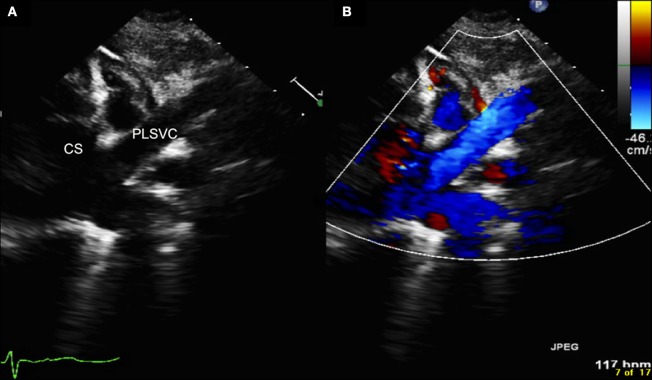
**Echocardiography (A = normal, B = with color Doppler) showing the persistent left superior vena cava draining into the dilated coronary sinus.** CS, coronary sinus; PLSVC, persistent left superior vena cava.

The most frequent manifestation of PLSVC is with drainage into the coronary sinus (Figure 1) (1–4), while drainage to the left atrium is extremely rare and always associated with complex congenital heart defects (1–4, 13, 15). Rarely, PLSVC is associated with absence or extreme hypoplasia of the right superior vena cava (1–4, 6, 12).

PLSVC can occur with the normal appearance of innominate vein (or left brachiocephalic vein), but in the majority of cases the innominate vein is absent (1–4).

The presence of PLSVC without innominate vein, has to be taken into account to plan the right venous cannulation for cardiopulmonary bypass and bypass management, and also the surgical approach when a cavo-pulmonary anastomosis is indicated for patients with functionally univentricular hearts (1–5, 7–9).

In this study the incidence of PLSVC was 13.5%, statistically higher (*P* < 0.0009) than the highest reported in the literature (4%) for pts with congenital heart defects (4), and observed in various types of congenital heart defects, suitable to either biventricular or univentricular type of repair (Table 2).

### Interruption of the inferior vena cava (IVC)

The IVC interruption is always associated with complex congenital heart defects, and the incidence has been reported in the literature between 0.15 and 1.3% (1, 4, 16).

**Figure 2:**
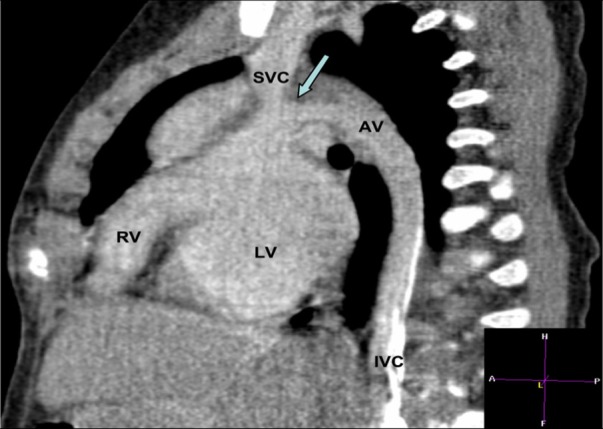
**CT angiography scan showing the continuation of the interrupted inferior vena cava into the azygos vein with a narrow connection (arrow) in a patient with hypoplastic right ventricle.** AV, azygos vein; IVC, inferior vena cava; LV, left ventricle; RV, right ventricle; SVC, superior vena cava.

Generally the interruption of IVC is accompanied by either azygos (Figure 2) or hemi-azygos continuation with the superior vena cava (1–4, 16, 28–35).

**Figure 3:**
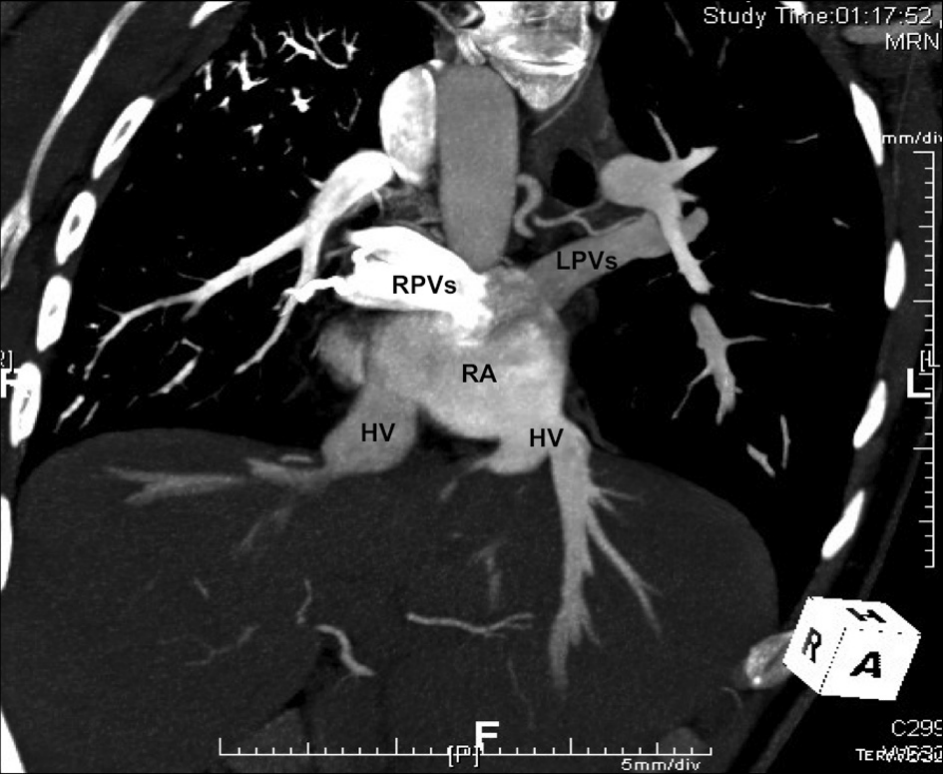
**CT angiography scan of a patient with left isomerism and interruption of the inferior vena cava, showing the presence of separate hepatic veins draining into the right atrium, and the presence of total anomalous pulmonary venous connection with “polarized” connection of the pulmonary veins to the right atrium, the right connected to the right side and the left to the left side.** HV, hepatic vein; LPVs, left pulmonary veins; RA, right atrium; RPVs, right pulmonary veins.

This systemic venous anomaly very often occurs in the presence of either right or left isomerism, with association of pulmonary venous anomalies (Figure 3) and complex intra-cardiac defects (1–4, 16, 28–35).

In patients with these complex heart malformations the surgical approach can rarely consider the plan toward a bi-ventricular type of repair (1, 2, 32, 34), and therefore complete and precise definition of the morphology of the systemic venous return is necessary for an appropriate planning of the surgical strategy to achieve a uni-ventricular type of repair (1, 2, 7, 9, 29–31, 33–35).

This study showed an incidence of IVC interruption in 3.2% of the pts, all with complex intra-cardiac morphology, definitely higher than the incidence reported in the literature (0.15–1.3%) (1, 4, 16), even if the difference did not reach statistical significance because of the relatively small number of patients. In our study all five patients with IVC interruption presented with simultaneous presence of partial or total anomalous pulmonary venous connection (Table 2).

### Retro-aortic innominate vein

The incidence of retro-aortic innominate (or left brachiocephalic) vein has been reported between 0.2 and 1.0% in the literature, generally in the presence of congenital heart defects (17–27).

In the vast majority of the reported cases the presence of retro-aortic innominate vein was associated with tetralogy of Fallot and right aortic arch (20, 21, 24, 25, 27).

The presence of retro-aortic innominate vein indicates modification of the cannulation of the superior vena cava to avoid obstruction to the drainage of the innominate vein during cardiopulmonary bypass.

Furthermore, this associated malformation can create practical difficulties when reconstruction of the pulmonary arteries is required in patients with tetralogy of Fallot and hypoplastic or stenotic pulmonary arteries, particularly in the presence of right aortic arch and dilated ascending aorta.

**Figure 4:**
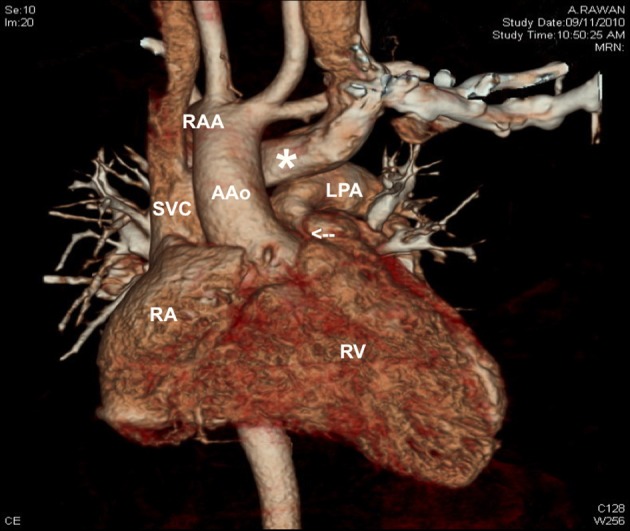
**CT angiography scan showing the presence of retro-aortic innominate vein (white asterisk), in a patient with tetralogy of Fallot with narrowed infundibulum (white arrow) and right aortic arch.** AAo, ascending aorta; LPA, left pulmonary artery; RAA, right aortic arch; RA, right atrium; RV, right ventricle; SVC, superior vena cava.

In our study the incidence of retro-aortic innominate vein was 1.9%, in all our patients (Table 2) associated with tetralogy of Fallot and right aortic arch (Figure 4).

With regard to the potential causes of the elevated incidence of systemic venous anomalies in our study, we can only offer speculations. All patients belong to the same Arab ethnicity, and this specific population presents from generations a strong social preference for consanguineous marriages. This has been proved to be associated with an elevated incidence of genetic anomalies, including congenital heart defects. Previous studies revealed an increased incidence of intra-cardiac congenital heart defects, but the incidence of systemic venous anomalies to the best of our knowledge has not been previously investigated (36–40).

Echocardiography generally identifies the presence of systemic venous anomalies in the pre-operative period, allowing an appropriate decision-making plan. The accuracy of the observation is related with the expertise and the associated anomalies.

In our study the elevated incidence of false negative echocardiography diagnosis (14/28 = 50%) can be attributed to the learning curve of a newly established unit, and the false negative diagnosis was valid for all types of systemic venous anomalies reviewed in this study (Table 1).

The use of complimentary investigations, like CT scan, MRI, or cardiac catheterization with angiography, can increase the percentage of pre-operative diagnosis in the presence of systemic venous anomalies.

In our experience a CT scan, performed in 8/28 (=28.6%) patients with systemic venous anomaly, after false negative diagnosis obtained with echocardiography, achieved in all complete and accurate diagnosis.

## Limits of the study

This study is limited by: (a) the fact that is a single center retrospective study; (b) the patients' selection criteria for the study population included only patients who underwent surgery for congenital heart defects, without any control group available; (c) the number of patients is relatively small.

## Conclusions

The incidence of systemic venous anomalies in Middle Eastern patients with congenital heart defects is undoubtedly higher than in the patients' population reported in the literature.

A careful pre-operative screening, using all the available methods of diagnostic investigation, should be performed in all patients with congenital heart defects from this region to better identify all systemic venous anomalies for a more accurate decision-making and surgical planning.

### Conflict of interest statement

The authors declare that the research was conducted in the absence of any commercial or financial relationships that could be construed as a potential conflict of interest.
